# Demographic and Clinical Characteristics of Retinoblastoma Cases at a Tertiary Care Center in Eastern India

**DOI:** 10.7759/cureus.64659

**Published:** 2024-07-16

**Authors:** Mobashir S Ali, Rajnee Sinha, Gyan Bhaskar, Neha Kumari, Bibhuti Sinha

**Affiliations:** 1 Ophthalmology, Indira Gandhi Institute of Medical Science, Patna, IND

**Keywords:** tumor, socioeconomic status, demography, malignancy, developing countries, retinoblastoma, squint, outcome, burden, leukocoria

## Abstract

Objective: This study aims to evaluate the burden, demographic profile, clinical characteristics, and management of retinoblastoma (RB) cases at a tertiary care center.

Material and methods: This was a hospital-based study conducted in a tertiary care center in the Department of Ophthalmology from January 2018 to December 2022. All referred and newly diagnosed cases of RB coming to the outpatient department were included in the study after obtaining written informed consent from parents or guardians. Data collected were analyzed in terms of demographic profile, socioeconomic status, and clinical characteristics of the disease at the time of presentation and its treatment.

Results: Out of 155,671 new patients seen in the outpatient eye department during the study period, 118 eyes of 94 patients were diagnosed with RB. The burden of disease was found to be 60.4 cases per 100,000 patients. Malignancy was unilateral in 74.47% and bilateral in 25.53% of cases. The male-to-female ratio was 1.7:1. The mean age at presentation was 30.86±19.5 months. A family history of RB was seen in 4.26% of cases. Of the patients, 80.85% belonged to upper-lower socioeconomic status. Most of the cases presented to us at an advanced stage of the disease (i.e., groups E and D).

Conclusion: Most of our cases present at an advanced stage of RB, resulting in poor outcomes and survival rates. It is necessary to organize awareness campaigns about the fatal nature of the disease so it can be diagnosed early, leading to better visual and survival outcomes.

## Introduction

Retinoblastoma (RB) is the most frequent intraocular neoplasm of childhood despite being a rare disease. The incidence of RB ranges from one in 15,000 to one in 18,000 live births globally, with 7,000-8,000 cases per annum [1]. The incidence rate varies from six cases per million in India to 11.8 cases per million worldwide in children below five years of age. The mean age at diagnosis of RB is 18 months. Bilateral cases are generally detected early, before 12 months of age, whereas unilateral cases generally present slightly later at around 24 months [2].

More than 60% of RB cases are unilateral and sporadic, whereas the remaining 40% are inherited, with both eyes involved in approximately 25%-35% of cases [2]. The World Health Organization reports that approximately 66% of children with RB are diagnosed before two years of age and 95% before five years of age. Leukocoria is the most common presenting feature of RB, which may also be associated with strabismus [3].

Based on data from several hospital-based cancer registries for two arbitrary 10-year time frames (1984-1993 (729 RB cases) and 1994-2003 (745 RB cases)), the Indian Council of Medical Research found a rise in RB cases [4]. The survival rate is low in developing countries, such as 48% in India and 20%-46% in African countries, as compared to developed countries such as the United States, where the survival rate is around 100% [5]. The increased mortality and visual disability in developing countries may be due to delays in diagnosis and inadequate healthcare facilities that can be attributed to poor socioeconomic status and lack of awareness about the severity of the disease [4].

If diagnosed early, not only the patient's life but also their eye and vision can potentially be saved by the use of local treatment in the form of laser photocoagulation, cryotherapy, transpupillary thermotherapy (TTT), and brachytherapy [3]. Drug delivery to targeted tissue in the form of intra-arterial and intravitreal chemotherapy are recent developments in RB management [3]. Multimodal therapy in the form of initial triple-drug high-dose chemotherapy (3-6 cycles) followed by surgery, orbital radiotherapy, and an additional 12-cycle standard-dose chemotherapy was developed as a standard treatment protocol by Honavar and Singh in 2005 [1].

However, unfortunately, most cases of RB in developing countries such as India are in an advanced stage at the time of presentation, where enucleation remains the only choice to attempt to save the patient's life [3]. With a multimodal approach, the disease is potentially curable if diagnosed in an early stage, and treatment must be individualized for each case [2]. Epidemiological studies conducted in different regions of India have reported that children with RB often present in an advanced stage for treatment. Lack of awareness about the fatal nature of the disease and poor patient compliance may be the main reasons for increased mortality in India [6]. As ours is a state tertiary care center with a dedicated team from the Department of Ophthalmology and a regional cancer center for retinoblastoma patients, most of our cases are referrals from all districts of Bihar and managed by a multidisciplinary approach.

To the best of our knowledge, there are no studies on the burden, demographic factors, clinical characteristics, and treatment of RB among the Bihar population. Therefore, the purpose of our study was to address this lacuna in the literature.

## Materials and methods

This was a hospital-based, observational, prospective study conducted at a tertiary care center during 2018-2022. The study was performed in accordance with the tenets of the Declaration of Helsinki. The study was approved by the Institutional Ethics Committee of Indira Gandhi Institute of Medical Science, Patna, prior to its start (approval number: 45/Acad). Referred and newly diagnosed cases of RB in the outpatient department of the eye and RB clinic were included in the study after obtaining written informed consent from parents or guardians. Those with other ocular malignant conditions such as rhabdomyosarcoma and leukemias, cases of leukocoria other than retinoblastoma, and those not willing to participate in the study were excluded. Demographic details, including age at presentation, laterality of the disease, presenting symptoms, and clinical signs in the form of leukocoria, proptosis, and strabismus, were noted for each patient. A detailed family history pertaining to the educational level and occupation of the head of the family, as well as household income, was collected from the parents. Families were scored using Kuppuswamy's Socioeconomic Status Scale on the basis of education, occupation, and family income per month into five categories (upper, upper middle, lower middle, upper lower, and lower) (Table 1) [7].

**Table 1:** Modified Kuppuswamy's Socioeconomic Status Scale 2019

Score	Socioeconomic class
26-29	Upper (I)
16-25	Upper middle (II)
11-15	Lower middle (III)
5-10	Upper lower (IV)
<5	Lower (V)

Each patient's ocular examination included visual acuity assessment, pupillary reaction, anterior segment examination by slit lamp, dilated fundus evaluation, and examination under general anesthesia, including intraocular pressure, laterality, and corneal diameter. Diagnosis was based on ophthalmological examination under anesthesia and radiological examination, which included ocular ultrasonography and magnetic resonance imaging of the brain and orbit for staging of the disease and to rule out extraocular spread. The International Classification of Intraocular Retinoblastoma and the International Retinoblastoma Staging System were used for the grouping and staging of the disease (Table 2 and Table 3) [4,8].

**Table 2:** International Classification of Intraocular Retinoblastoma

Group	Clinical features	
Group A		Tumor ≤ 3 mm in size and located >3 mm from the foveola and >1.5 mm from the optic disc
Group B		Tumor > 3 mm in size and located ≤3 mm from the foveola and ≤1.5 mm from the disc with clear subretinal fluid ≤ 3 mm from the tumor margin
Group C		Focal seeds (≤3 mm both vitreous and subretinal seeds from the tumor)/discrete RB with/without subretinal fluid ≤ 1 quadrant of the retina
Group D		Diffuse seeds (>3 mm both vitreous and subretinal seeds from the tumor)/RB with/without subretinal fluid ≥ 1 quadrant
Group E		Extensive retinoblastoma occupying >50% of the globe or touching the lens, opaque media from hemorrhage in the anterior chamber, vitreous, or subretinal space, neovascular glaucoma, invasion of the post-laminar optic nerve, choroid (>2 mm), sclera, orbit, anterior chamber

**Table 3:** International Retinoblastoma Staging System CNS: central nervous system

Stage	Clinical description
Stage 0	No enucleation (one or both eyes may have an intraocular disease)
Stage I	Eye enucleated, completely resected histologically
Stage II	Eye enucleated, microscopic residual tumor
Stage III	Regional extension: (a) overt orbital disease and (b) preauricular or cervical lymph node extension
Stage IV	Metastatic disease: (a) hematogenous metastasis (single lesion and multiple lesion) and (b) CNS extension (prechiasmatic lesion, CNS mass, and leptomeningeal disease)

In addition to the ophthalmological examination, a general physical examination and a lymph node assessment were performed in all cases. Liver function tests, kidney function tests, and complete blood count were done routinely. Metastatic workup with chest X-ray, ultrasound of the abdomen, bone marrow biopsy, and cerebrospinal fluid examination were also conducted in suspected cases of metastasis.

As there is no one proven definitive therapy for orbital RB, management was individualized for each RB case. Intraocular unilateral cases in groups A and B were managed with laser photocoagulation and cryotherapy, respectively, in the form of local treatment, which were very few, as they were diagnosed accidentally during examination under anesthesia. For those with advanced unilateral intraocular disease, enucleation was given to patients as a first-line treatment. For all bilateral cases and extraocular spread, neoadjuvant chemotherapy was given as a first-line treatment, followed by enucleation/exenteration of the worse eye, and conservative treatment in the form of cryotherapy or laser photocoagulation given to the better eye in cases of intraocular disease. For cases of extraocular spread, following 2-3 cycles of neoadjuvant chemotherapy, enucleation was done, followed by external beam radiation and adjuvant chemotherapy for an additional 6-10 cycles [4,8]. Enucleated eyeballs were sent for histopathological examination. TTT patients were referred to higher-level centers, as our center does not have this facility. Post-enucleation patients were asked to follow up and periodically were examined for any regression or progression of the tumor in the other eye in cases of bilateral intraocular disease or any recurrence of the tumor. After the completion of treatment, follow-up was done at three-month intervals for one year, followed by an examination every 4-6 months for three years or until the child reached six years of age and yearly thereafter [4].

Statistical analysis

Data were recorded on a predesigned proforma and were managed on a spreadsheet using the Microsoft Excel 2007 software (Microsoft Corp., Redmond, WA). Descriptive statistics included mean, standard deviation, percentage, and ratio. Statistical analysis was done using SPSS (IBM SPSS Statistics for Windows, version 20.0; Armonk, NY). Categorical variables were compared using the χ2 test. A p-value of <0.05 was considered statistically significant.

## Results

**Figure 1:**
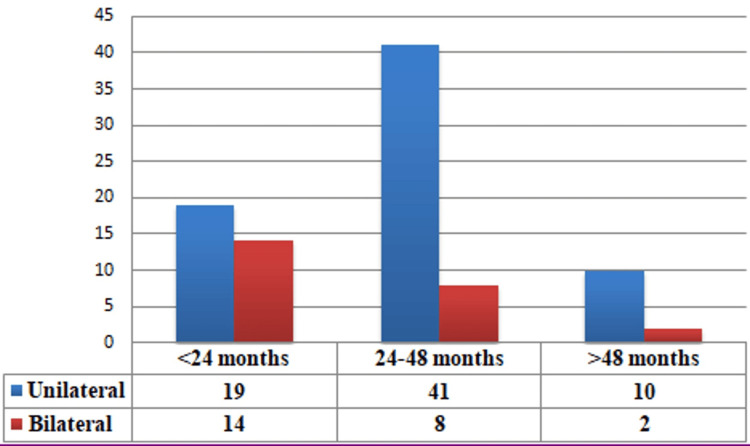
Age-wise distribution of cases at presentation

Out of 155,671 new patients seen at the outpatient department of ophthalmology in a tertiary care center, 118 eyes of 94 patients were identified to have RB, resulting in a burden of disease of 60.4 per 100,000 patients. Sixty (63.83%) patients were male, and 34 (36.17%) were female, resulting in a male-to-female ratio of 1.7:1. The disease was unilateral in 70 (74.47%) patients and bilateral in 24 (25.53%) patients. Eighty-two (87.23%) patients were under 48 months of age, and 12 (12.77%) patients were above 48 months of age, as shown in Figure 1.

The mean age at presentation was 30.86±19.5 months for all cases, with that of unilateral cases being 32.88±20.52 months and that of bilateral cases being 26.64±13.2 months. Although the mean age of presentation in the bilateral group was lower than in the unilateral group, the difference was not statistically significant (p=0.489). The demographic information of the 94 cases is presented in Table 4.

**Table 4:** Demographic distribution of retinoblastoma cases

Variable	Number of cases (n=94)
Age at presentation	
<24 months	33 (35.11%)
24-48 months	49 (52.13%)
>48 months	12 (12.77%)
Mean age at presentation (months)	30.86±19.5
Sex	
Male	60 (63.83%)
Female	34 (36.17%)
Laterality	
Unilateral	70 (74.47%)
Bilateral	24 (25.53%)
Socioeconomic status	
Upper lower	76 (80.85%)
Lower middle	9 (9.57%)
Upper middle	9 (9.57%)
Family history of retinoblastoma	4 (4.26%)
History of consanguinity	4 (4.26%)

A family history of RB or other cancers was found in four (4.26%) and six (6.38%) children, respectively. A history of consanguinity was found in four (4.26%) cases.

The burden of the disease was calculated to be 60.4 per 100,000 patients according to the following formula: burden (percentage) of disease = total number of retinoblastoma cases from January 2018 to December 2022 / total number of patients coming to the OPD from January 2018 to December 2022 x 100.

The most common presenting symptom was leukocoria, which was seen in 65 (69.14%) cases, followed by proptosis in 25 (26.60%) cases and squint associated with leukocoria in four (4.26%) cases.

Most of the tumors (n=69; 73.40%) were located intraocularly, and 25 (26.60%) patients had extraocular tumor extension at presentation. Bilateral cases were six (24%) with an average age of one year, and unilateral cases were 19 (76%) with an average age of four years. Optic nerve involvement was seen on magnetic resonance imaging in 12 (12.77%) eyes. A total of 4, 8, 7, 17, and 82 eyes were categorized as groups A, B, C, D, and E, respectively, which means that most of the patients included in the study had advanced disease at presentation (Table 5).

**Table 5:** Grouping of RB cases RB: retinoblastoma

Group	Number of eyes (n=118)
A	4 (3.39%)
B	8 (6.78%)
C	7 (5.93%)
D	17 (14.4%)
E	82 (69.50%)
Total	118

Overall, 4, 42, 6, 22, and 3 eyes presented in stages 0, I, II, III, and IV, respectively, after excluding those who refused treatment and went to higher centers (Table 6).

**Table 6:** Staging of the disease

Stage	Number of eyes (n=77)
0	4 (5.19%)
I	42 (54.55%)
II	6 (7.80%)
III	22 (28.58%)
IV	3 (3.90%)

Metastasis was present in three (3.20%) cases. Two (66.67%) cases were unilateral of age group 2 and 5 years, respectively, and one (33.3%) case presented bilaterally of age group 2.

Regarding treatment history, out of 94 children included in the study, 12 (12.77%) refused treatment, 15 (15.96%) were lost to follow-up, and 15 (15.96%) were referred to a higher center for further management either at the patient's request or for TTT or intravitreal chemotherapy. Of the remaining 52 patients who underwent treatment at our center, in advanced bilateral cases, 48 (51.06%) patients were given neoadjuvant chemotherapy, which was followed by enucleation in 48 (51.06%) and exenteration in four (4.26%) extraocular cases, and subsequent adjuvant chemotherapy and radiation therapy as needed (Table 7).

**Table 7:** Treatment history

Variable	Number of cases (n=94)
Refused treatment	12 (12.77%)
Lost to follow-up	15 (15.96%)
Referred to a higher center	15 (15.96%)
Neoadjuvant chemotherapy	48 (51.06%)
Enucleation	48 (51.06%)
Exenteration	4 (4.26%)
Recurrence	3 (3.19%)

Enucleated eyeballs were sent for histopathological examination. Among those who received treatment, recurrence occurred in three (3.19%) cases, and death occurred in 30 (31.91%) cases in the four-year follow-up period. In stage IV, the death rate was 100% despite treatment, whereas in up to stage III, the success rate was 90%. Survival rate analysis was not done as mentioned in the limitations.

## Discussion

RB is a rare pediatric ocular malignancy in early childhood; as its incidence is low compared to other ocular diseases, children are not regularly assessed for it. In developing nations such as India, there are more cases of RB with a late presentation, which may be due to a lack of awareness about the consequences of the disease and proper healthcare facilities in the periphery [6]. Therefore, identifying the disease by its clinical features on presentation becomes very important for the patient's long-term prognosis [8]. Several studies have been conducted to investigate the epidemiological pattern of RB in different parts of the world and different regions of India, but to the best of our knowledge, there are no studies from Bihar on the burden, demographic profile, clinical characteristics, and treatment of this disease.

In our study, out of 155,671 new patients who reported to the OPD of our tertiary care center, 94 (118 eyes) had RB, giving a relative percentage (i.e., burden of disease) of 0.06, or 60.4 RB patients per 100,000 patients seen, which is a much lower frequency when compared to other diseases of the eye. Our findings were similar to those of a study conducted by Subha et al. [9], in which the incidence of RB was much lower compared to other diseases of the eye.

Our study comprised 60 (63.83%) male patients and 34 (36.17%) female patients, which was comparable to studies by Singh et al. [4], Kabre et al. [10], and Kaimbo et al. [11]. However, in a study by Subha et al. [9], sex had little bearing on RB frequency. The male-to-female ratio in this study was 1.7:1, which is slightly higher when compared to studies done by Hazarika et al. [2], Kaimbo et al. [11], and Sahu et al. [12], which had male-to-female ratios of 1.2:1, 1.9:1, and 1.4:1, respectively. The majority of our patients being male may be because ours is an underdeveloped state where people are more worried about male children and prefer bringing them for treatment over female children. Thus, female children often become victims of neglect, suggesting that parents are more likely to seek tertiary care services for males than for females.

Of the 94 cases, 24 (25.53%) had bilateral involvement, and 70 (74.47%) had unilateral involvement, which was similar to studies done by Subha et al. [9] and Sahu et al. [12].

In this study, the mean age of diagnosis of RB was 30.86±19.5 months. The mean age of diagnosis in unilateral RB was 32.88±20.52 months and that of bilateral RB was 26.64±13.2 months. The mean age of diagnosis in this study was comparable with that of other studies, as shown in Table 8 [4,8,13-17].

**Table 8:** Comparison of mean age of presentation in different regions PGIMER: Postgraduate Institute of Medical Education and Research, LVPEI: L V Prasad Eye Institute

Region	Mean age of diagnosis of RB (months)	Mean age of diagnosis of unilateral cases of RB (months)	Mean age of diagnosis of bilateral cases of RB (months)
PGIMER, India [4]	34.7	36.5	30.9
China [13]	23	27	15
Malaysia [14]	22	29	14
Pakistan [15]	35.98	39	31
Brazil [16]	-	33.8	19.15
*LVPEI*, India [8]	29	-	-
Kolkata, India [17]	-	36.2	23.5
Bihar (our study)	30.86	32.88	26.64

A family history of RB and other cancers was found in four (4.26%) and six (6.38%) children, respectively, similar to studies done by Hazarika et al. [2] and Sahu et al. [12].

A history of consanguinity was present in three (3.19%) cases, of which two (66.67%) presented bilaterally and one (33.33%) presented unilaterally (p=0.03). Based on these results, a close correlation existed between consanguinity and bilateral involvement. These results were in contrast with those of the studies by Subha et al. [9] and Sahu et al. [12], who reported consanguinity history in 23% and 17% of cases, respectively.

**Figure 2:**
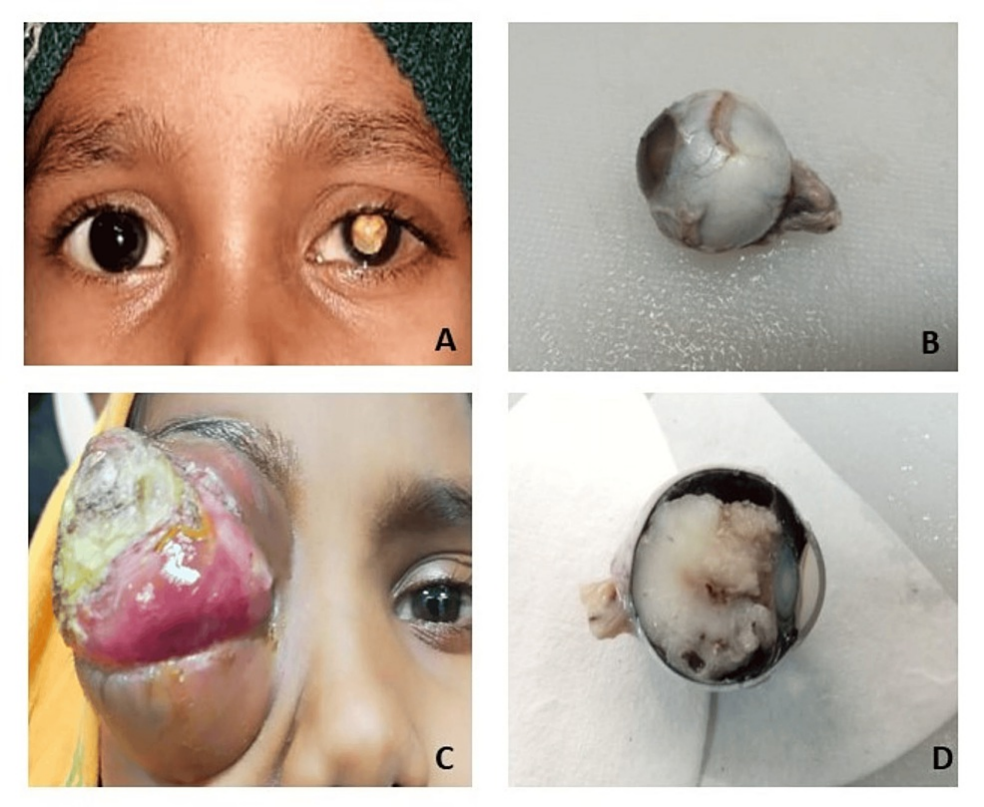
Clinical characteristics and enucleated eyeball specimen showing retinoblastoma tumor (A) Leukocoria, (B) enucleated eyeball, (C) proptosis, and (D) cut section of enucleated eyeball

In our study, leukocoria was the most common presenting symptom, followed by squint and proptosis, as seen in Figure 2. Rare findings of microphthalmos, hyphema, and secondary glaucoma were similar to a study done by Balasubramanya et al. [18].

In our study, 76 (80.85%) patients belonged to upper-lower socioeconomic status, which is similar to a study done by Bhattacharya et al. [17], where 80% of families belonged to the lower, upper-lower, or lower-middle strata of society according to the Kuppuswamy Classification Scale [4]. Regarding the socioeconomic status of patients suffering from this rare disease, it has been observed to be more prevalent in economically poorer parts of the world, being as high as 42.5 per million (i.e., the incidence rate of RB for children aged 0-4 years) in Mali, Africa, as compared to 3.5 per million in Bulgaria [17].

**Figure 3:**
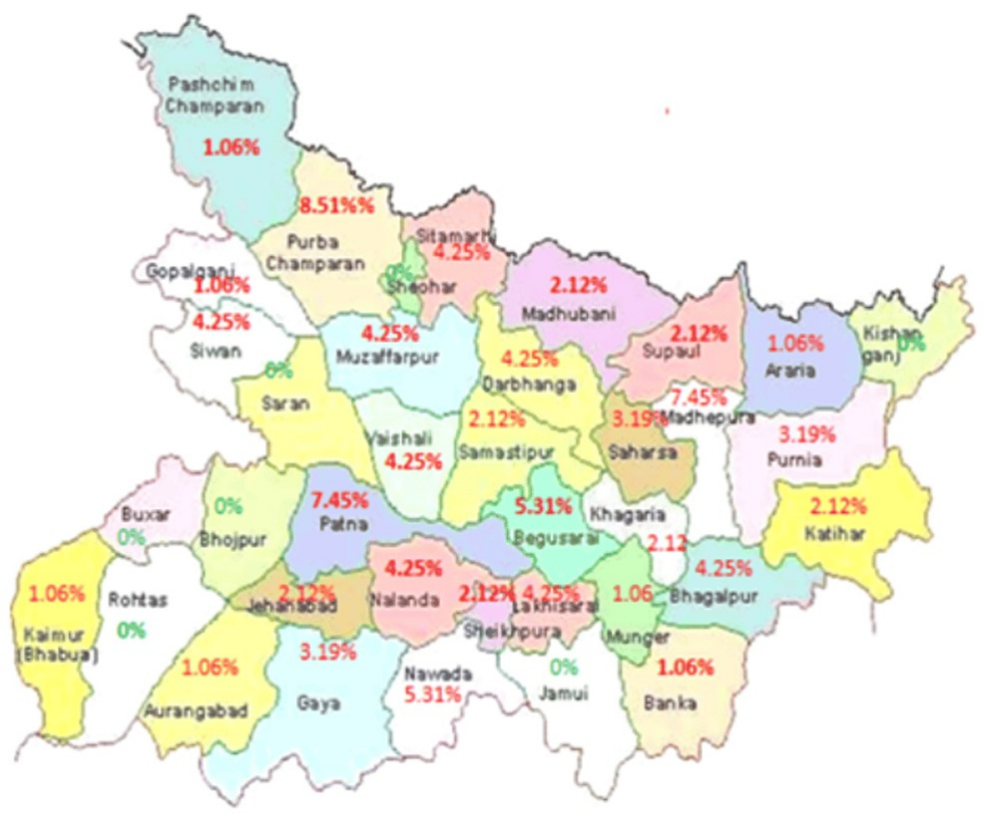
District-wise distribution of retinoblastoma cases in Bihar

In our study, most patients reported being from North Bihar, as shown in Figure 3, which may be because patients from South Bihar might be traveling directly to other higher-level centers, leading to referral bias of RB patients.

Study limitations

This study had some limitations. Incidence and prevalence were not calculated, as this was a single-center study. In addition, as some patients were lost to follow-up and some went to higher centers for further opinion, survival rate analysis was not done. Finally, genetic analysis of patients was not done.

## Conclusions

The majority of RB patients belonged to upper-lower socioeconomic status with late presentation of the disease, which may result in poor visual and survival outcomes. Late presentation may be due to a lack of awareness about the consequences of the disease and inaccessibility to proper medical facilities, which may limit achieving high cure rates. This hurdle can be overcome by organizing awareness campaigns, which can target educating the public and healthcare professionals at different rural and urban healthcare centers, as well as educating patients during vaccination appointments about early signs of RB and its complications. Ocular examination at the time of immunization can be also promoted to look for any white reflex. Such awareness programs can be highly effective in limiting the burden of RB, especially among those with lower socioeconomic status where greater attention is required.
